# Assessment of Pulmonary Circulation of Critically Ill Patients Based on Critical Care Ultrasound

**DOI:** 10.3390/jcm13030722

**Published:** 2024-01-26

**Authors:** Shiyi Gong, Xin Ding, Xiaoting Wang

**Affiliations:** Department of Critical Care Medicine, Peking Union Medical College Hospital, Beijing 100730, China; gongshiyi@pumch.cn

**Keywords:** critical illness, point-of-care ultrasound, pulmonary circulation

## Abstract

Pulmonary circulation is crucial in the human circulatory system, facilitating the oxygenation of blood as it moves from the right heart to the lungs and then to the left heart. However, during critical illness, pulmonary microcirculation can be vulnerable to both intrapulmonary and extrapulmonary injuries. To assess these potential injuries in critically ill patients, critical point-of-care ultrasound can be used to quantitatively and qualitatively evaluate the right atrium, right ventricle, pulmonary artery, lung, pulmonary vein, and left atrium along the direction of blood flow. This assessment is particularly valuable for common ICU diseases such as acute respiratory distress syndrome (ARDS), sepsis, pulmonary hypertension, and cardiogenic pulmonary edema. It has significant potential for diagnosing and treating these conditions in critical care medicine.

## 1. Introduction

Pulmonary circulation is a crucial component of the circulatory system. It involves the right ventricle pumping hypoxic blood into the pulmonary artery, where it undergoes gas exchange in the pulmonary capillaries before flowing into the left atrium to continue systemic circulation [1]. Despite historically being overlooked due to limited evaluation methods, the importance of pulmonary circulation in critically ill patients, particularly in conditions like acute respiratory distress syndrome (ARDS), sepsis, pulmonary hypertension, and cardiogenic pulmonary edema, has gained recognition. Early detection and intervention for pulmonary circulation injury can have a significant impact on patient prognosis.

With the significant advancement of critical ultrasound, bedside assessment of pulmonary circulation has become feasible. Critical point-of-care ultrasound enables both qualitative and quantitative evaluation of the right atrium, right ventricle, pulmonary artery, pulmonary vein, and left atrium along the direction of pulmonary blood flow while also allowing for the identification of pulmonary microcirculation injury. This article will comprehensively review pulmonary circulation injuries in critically ill patients and provide detailed insights into the methods of evaluating pulmonary circulation using critical ultrasound.

## 2. The Characteristics of Pulmonary Circulation

### 2.1. Vulnerability of Pulmonary Circulation

Pulmonary circulation comprises the pulmonary arterial tree, capillary bed, and venous tree, facilitating the exchange of gases in the lungs. The pulmonary artery, with thin walls and numerous branches, forms a low-pressure system. At the same time, the pulmonary microcirculation system contains a huge number of capillary networks to guarantee the efficiency of the gas exchange process. Additionally, the pulmonary vein, characterized by low pressure and large volume, plays a crucial role in transporting oxygen blood to the left heart.

The unique anatomical features of pulmonary circulation predispose critically ill patients to pulmonary circulation injury. The thinner and less elastic walls of the pulmonary artery and right ventricle make the pulmonary artery more sensitive to changes in pressure load. Increased pulmonary circulatory resistance resulting from factors such as hypoxemia, mechanical ventilation, or left heart disease can lead to the development of pulmonary hypertension and right heart dysfunction. Additionally, the low pressure in the pulmonary veins means that an increase in left atrial pressure will correspondingly raise the pressure in the pulmonary veins and capillaries. According to Starling’s law, the fluid in the pulmonary capillaries may leak into the pulmonary parenchyma and accumulate in the gas–blood barrier, which hinders the pulmonary gas exchange process. The hypoxemia and hypercapnia caused by pulmonary edema may cause pulmonary arteries constriction and exacerbate right heart dysfunction [2,3,4].

In critically ill patients, right ventricular dysfunction originates from primary or secondary lung injury due to various etiologies. Following invasive mechanical ventilation, inflation of the lungs and increased pulmonary vascular resistance further elevate right heart afterload. Pulmonary hypertension develops as a consequence of both primary and secondary lung injury as well as inappropriate mechanical ventilation settings. As the disease progresses, pressure overload and volume overload manifest in the right heart. Bedside ultrasound enables observation of acute cor pulmonale (ACP) manifestations such as right heart enlargement and decreased right heart motion. Clinically, an increase in central venous pressure (CVP) serves as the most direct manifestation of ACP, while a decrease in cardiac output (CO) reflects hemodynamic collapse caused by ACP.

### 2.2. Pulmonary Microcirculation Injury

Critically ill patients can suffer from pulmonary microcirculation injury, which, depending on the cause of the injury, can be broadly classified into two phenotypes. One phenotype is characterized by direct harm to alveolar epithelial cells, while the other is distinguished by damage to pulmonary capillary endothelial cells. 

COVID-19 pneumonia is an example of pulmonary microcirculation injury caused by epithelial damage. Once the virus enters the lungs, it directly attacks alveolar epithelial cells, leading to severe respiratory distress and hypoxemia. In severe cases, mechanical ventilation may be required as a lifesaving treatment. Furthermore, the viral infection can trigger a cytokine storm that not only harms the epithelial cells but also damages the pulmonary capillary endothelial cells, ultimately causing direct damage to the lungs themselves [5,6,7]. 

Sepsis is an example of pulmonary microcirculation injury caused by endothelial damage. This serious condition triggers a systemic inflammatory response, leading to the release of numerous inflammatory cytokines into the bloodstream. These cytokines attack the pulmonary capillary endothelial cells, causing damage to the air–blood barrier and increasing capillary permeability. This results in pulmonary interstitial edema and further damage to alveolar epithelial cells, ultimately exacerbating the patient’s hypoxia [8,9,10]. In this scenario, a pulmonary circulation lesion is a secondary injury caused by the critical illness. 

Both phenotypes ultimately result in damage to both alveolar epithelial and vascular endothelial cells, leading to increased leakage and typical pulmonary lesions associated with gravity-dependent ARDS. It is evident that pulmonary circulation exhibits distinct characteristics compared to the systemic circulation. Firstly, pulmonary circulation exhibits low resistance and is highly sensitive to pressure changes, making it susceptible to increases in pulmonary circulation resistance triggered by various factors. Secondly, pulmonary circulation experiences high blood flow, and increases in venous pressure or changes in permeability can lead to pulmonary edema. Thirdly, the pulmonary microcirculation is particularly vulnerable, as both direct lung damage and exogenous insults can impact the pulmonary microcirculation, thereby promoting lung disease and exacerbating pulmonary circulation deterioration. Consequently, it is crucial to assess the structure and function of pulmonary circulation and associated systems in critically ill patients.

## 3. Ultrasound Assessment of Pulmonary Circulation

**Figure 1:**
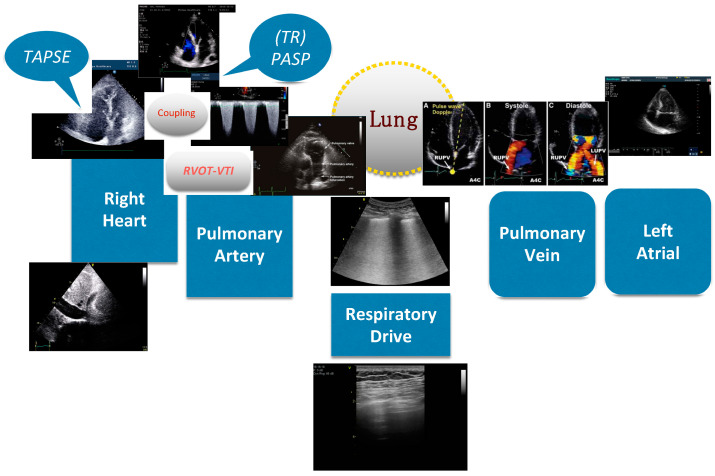
The anatomy and important ultrasound evaluation indicators of pulmonary circulation. Abbreviations: TAPSE, tricuspid annular plane systolic excursion; RVOT, right ventricular outflow tract; VTI, velocity-time integral; PASP, pulmonary artery systolic pressure; TR, tricuspid regurgitation.

Although pulmonary artery catheterization (PAC) can directly measure multiple indicators such as pulmonary artery pressure, cardiac output, and pulmonary capillary wedge pressure and is still considered the gold standard for evaluating pulmonary circulation. As an invasive way of pulmonary circulation evaluation, PAC is used in some selected cases. In contrast, critical care ultrasound can evaluate pulmonary circulation and related organs such as the heart, blood vessels, lungs, and diaphragm from multiple dimensions. Point-of-care ultrasound can be routinely used for pulmonary circulation assessment for critically ill patients. The anatomy of pulmonary circulation and important bedside ultrasound evaluation indicators are shown in Figure 1.

### 3.1. Cardiac Ultrasound Pulmonary Circulation Assessment

#### 3.1.1. Right Atrium

The right atrial pressure (RAP) can reflect the volume of venous return and right ventricular preload. In ICU, the right atrial pressure can be measured by the central venous pressure (CVP) through the central venous catheter (CVC). An elevated CVP is an independent risk factor for poor prognosis [11,12]. Meanwhile, we can indirectly estimate right atrial pressure based on the morphological assessment of the inferior vena cava (IVC) by point-of-care ultrasound. To accurately estimate right atrial pressure, we recommend utilizing point-of-care ultrasound to assess the inferior vena cava (IVC) morphology. At the subxiphoid view, the diameter of the IVC should be measured 2 cm from the opening of the right atrium [13,14]. Fixed and dilated IVC indicates an increase in right atrial pressure. The detailed method of right atrial pressure prediction based on IVC diameter and respiratory collapse is shown in Table 1. For ventilated patients, an IVC diameter of ≤12 mm often indicates that the right atrial pressure is not greater than 10 mmHg [15]. Zhang et al. found the ratio of transverse maximum and minimum IVC diameter greater than 1.7 can predict CVP less than 8 mmHg [16]. When assessing right atrial pressure by IVC ultrasound measurement, it is crucial to consider the impact of factors like mechanical ventilation, intra-abdominal pressure, and pericardial effusion. Direct CVP measurement remains the most accurate method, but in clinical settings, ultrasound assessment of the IVC can provide valuable information. However, it is essential to recognize these indirect methods’ limitations and potential confounders. 

**Figure 2:**
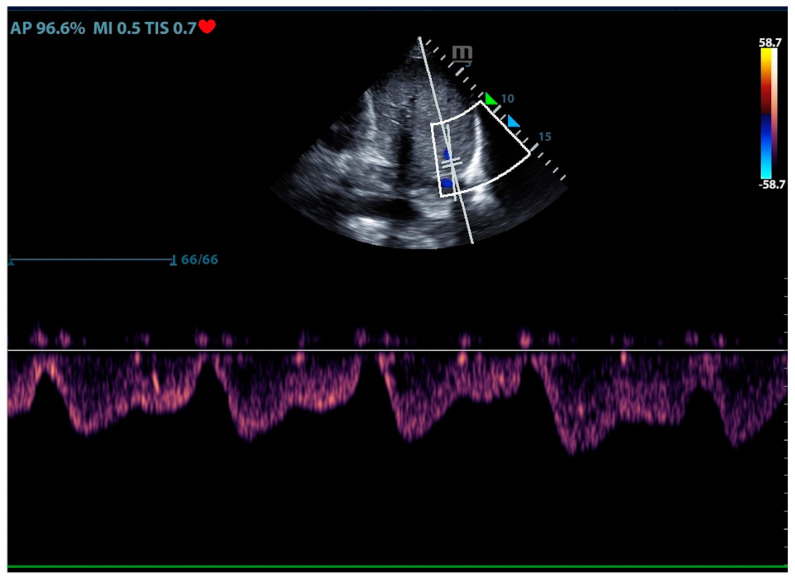
The ultrasound sign of hepatic venous excess waveform.

The Doppler ultrasound measured venous waveform can indirectly indicate the congestion level of the venous system and the pressure of the vena cava and right atrium. William et al. developed a venous excess ultrasound (VE×US) grading system based on the Doppler waveform of the hepatic vein, portal vein, and intra-renal vein. The severe VE×US grade indicated high CVP and congestion of the venous system [17]. The hepatic vein filling fraction, calculated from the maximum velocity of the S wave and D wave in the hepatic vein waveform, has been shown to correlate with elevated CVP when exceeding 55% [18]. (Figure 2) The use of venous excess ultrasound in clinical settings requires adequate training and experience for ICU physicians.

#### 3.1.2. Right Ventricle

The right ventricle (RV) is the starting point of pulmonary circulation. The function of the right ventricle is closely related to the resistance of pulmonary circulation. On the one hand, pulmonary circulation resistance is the determining factor of right ventricular afterload, which has a significant impact on the ejection of the right ventricle. On the other hand, the decrease in the contraction function of the right ventricle itself will also significantly affect the blood flow of pulmonary circulation.

**Figure 3:**
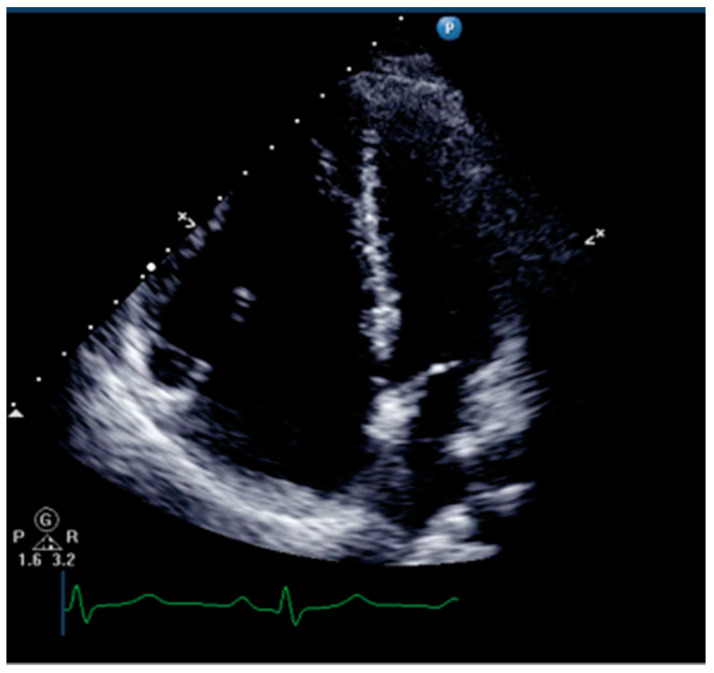
The ultrasound sign of right ventricular dilation.

Right ventricular dilatation and paradoxical movement of the interventricular septum are common ultrasound signs of right ventricular dysfunction for qualitative assessment of RV (Figure 3). Physicians can measure the ratio of right ventricular to left ventricular end-diastolic volume in the apical four-chamber view. The RV/LV area ratio over 0.6 indicates right ventricular enlargement, while over 1.0 indicates severe enlargement [14]. 

Paradoxical movement of the ventricular septum is a sign of increased RV preload and afterload, especially afterload. The leftward shift of the interventricular septum can be observed in the short-axis view, causing the left ventricle to be compressed into a “D” shape [14]. It should be noted that not only chronic pulmonary hypertension can cause right ventricular dilation, but when pulmonary artery pressure suddenly increases, the right ventricle can also acutely enlarge. 

The size of the right atrium and the thickness of the right ventricular free wall can help distinguish between acute and chronic right heart failure. Right atrium enlargement is often observed in patients with chronic pulmonary hypertension [14]. When the pulmonary artery pressure increases acutely, there is usually no significant right atrium enlargement. The thickness of the right ventricular free wall is usually measured during diastole in the subxiphoid four-chamber view, with a normal thickness of 3–5 mm [19], while in chronic pulmonary arterial hypertension, it can reach 10–11 mm [14].

**Figure 4:**
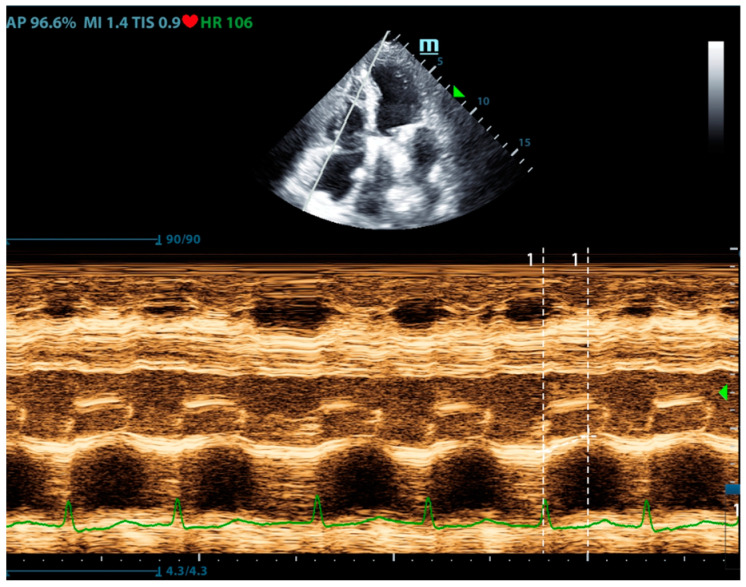
The ultrasound measurement of TAPSE.

Right ventricular systolic function is an important part of pulmonary circulation assessment. Compared with the left ventricle, which mainly performs a concentric and twisting motion, the contraction of the right ventricle is mainly a long-axis contraction. Therefore, the assessment of right ventricular long-axis systolic function can reflect the overall right ventricular systolic function. Tricuspid annular plane systolic excursion (TAPSE) is the most used indicator to reflect the long-axis contraction of the right ventricle (Figure 4). A TAPSE less than 16 mm indicates right ventricular systolic dysfunction [20]. Although it reflects the longitudinal systolic function, it is well correlated with other indicators that reflect right ventricular systolic function (such as RVEF and RVFAC) [21,22]. Comparing these parameters, TAPSE is most widely used in right heart systolic assessment for its feasibility and reliability. In addition, the systolic peak flow velocity S’ of the right ventricular free wall can also reflect the systolic function of the right ventricle. Tissue Doppler measurement is performed at the base of the right ventricular free wall, and S’ < 10 cm/s indicates right ventricular systolic dysfunction [23,24]. However, this method has poor measurement repeatability for segmental dysfunction and lacks reference values for all age groups, especially the elderly, so it was currently used as a research tool. 

In the long-axis view of the pulmonary artery, physicians can measure the velocity-time integral (VTI) of the right ventricular outflow tract (RVOT). As well as LVOT-VTI, RVOT-VTI is strongly associated with stroke volume and can predict low cardiac output and poor outcomes [25]. The use of RVOT-VTI as a surrogate indicator for cardiac output evaluation by point-of-care ultrasound can be considered in cases where accurately measuring LVOT-VTI is challenging (e.g., patients with aortic valve disease). However, obtaining the necessary views and appropriate measurement angle requires experience; this limits the application of RVOT-VTI in critical care and emergency settings.

#### 3.1.3. Pulmonary Artery

**Figure 5:**
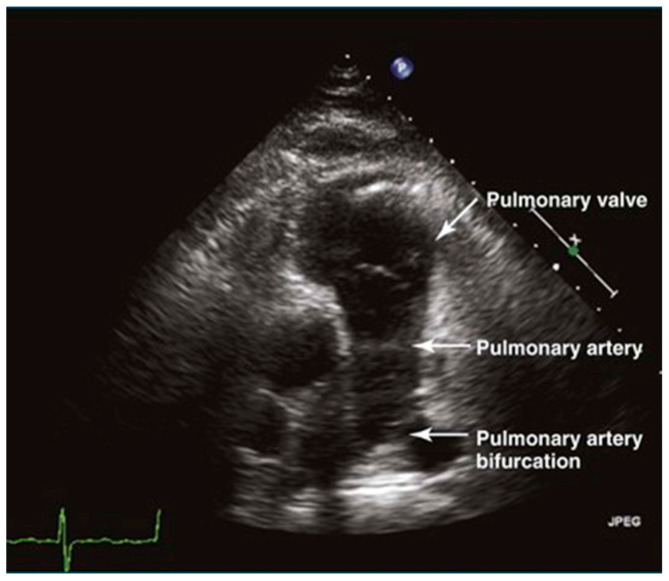
The sign of pulmonary artery in long-axis view.

The long-axis view of the pulmonary artery shows the main pulmonary artery and its branches, aiding in the identification of space-occupying lesions like thrombi (Figure 5). This view is crucial for diagnosing acute pulmonary embolism. Additionally, the width of the main pulmonary artery helps differentiate between acute and chronic pulmonary hypertension, with significant widening indicating the chronic form [14].

**Figure 6:**
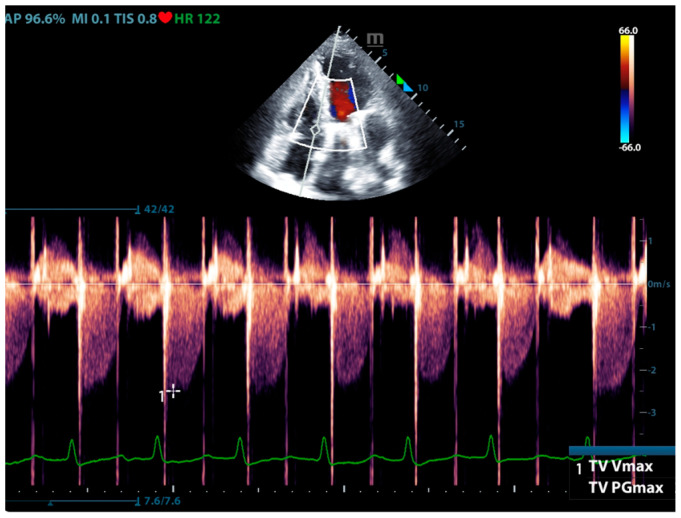
The ultrasound measurement of the maximum velocity of tricuspid valve regurgitation.

**Figure 7:**
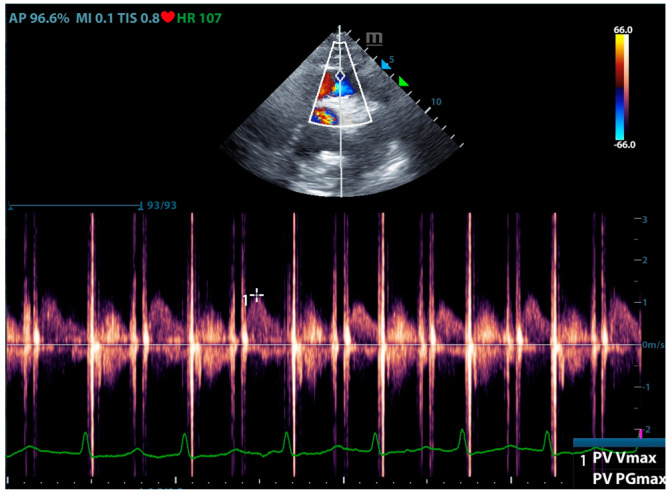
The ultrasound measurement of the maximum velocity of the pulmonary artery valve regurgitation.

Physicians can assess pulmonary artery pressure quantitatively with point-of-care ultrasound. According to Bernoulli’s principle, the systolic pressure and diastolic pulmonary artery pressure can be estimated, respectively, by measuring the maximum regurgitation velocity of the tricuspid valve and the pulmonary valve. The maximum regurgitation velocity of the tricuspid valve (TVR V_max_) can be measured in the apical four-chamber view (Figure 6), and the maximum velocity of the pulmonary artery valve regurgitation (PVR V_max_) can be measured in the pulmonary artery long-axis view (Figure 7). Physicians can estimate pulmonary artery systolic pressure (PASP), diastolic pressure (PADP), and mean pulmonary artery pressure (mPAP) based on the ultrasound-measured TVR V_max_ and PVR V_max_ by the following formulas:

PASP = TVR V_max_^2^ + RAP(CVP)

PADP= PVR V_max_^2^ + RAP(CVP)

mPAP = 1/3 ∗ PASP + 2/3 ∗ PADP

This can not only be used for the diagnosis of pulmonary hypertension but also for continuous and dynamic evaluation of pulmonary artery pressure [26]. It should be noted that the use of tricuspid regurgitation velocity to estimate pulmonary artery pressure with ultrasound Doppler has limitations. When the patient has severe tricuspid valve disease or severe right heart dysfunction, using the maximum tricuspid regurgitation velocity may underestimate the pulmonary artery pressure. Therefore, the evaluation of pulmonary hypertension should be combined with other ultrasound signs of the right ventricle, pulmonary artery, inferior vena cava, and right atrium.

#### 3.1.4. Right Ventricular-Pulmonary Arterial Coupling

Ventricular-arterial coupling refers to the relationship between myocardial contractility and ventricular afterload. Elevated pulmonary artery pressure in patients leads to increased right ventricular afterload, and in the initial stages of the disease course, right ventricular contractility increases to compensate for the increased right ventricular afterload. But with the further increase in right ventricular afterload, the right ventricle begins to enlarge and the contractility of the myocardium decreases. From the perspective of ventricular-arterial coupling, right ventricular enlargement has indicated that ventricular function is in a decompensated state, and right ventricular failure occurs [27]. With the further deterioration of right ventricular systolic function, there is a decline in right ventricular cardiac output. At the same time, the severe expansion of the right heart compresses the left heart, which can lead to a decrease in the cardiac output of the left ventricle. Decreased cardiac output can lead to lack of blood perfusion of tissues and organs, which can be life-threatening in severe conditions. This phenomenon that changes in one ventricle directly affect the function of the other ventricle is called ventricle-ventricular coupling. Right ventricle-pulmonary artery coupling can be quantitatively evaluated by measuring the ratio of TAPSE and PASP by ultrasound [28]. Previous studies have found that ultrasound measurement of a TAPSE/PASP ratio < 0.5 mm/mmHg is significantly associated with increased mortality in patients with sepsis [29].

#### 3.1.5. Pulmonary Vein

**Figure 8:**
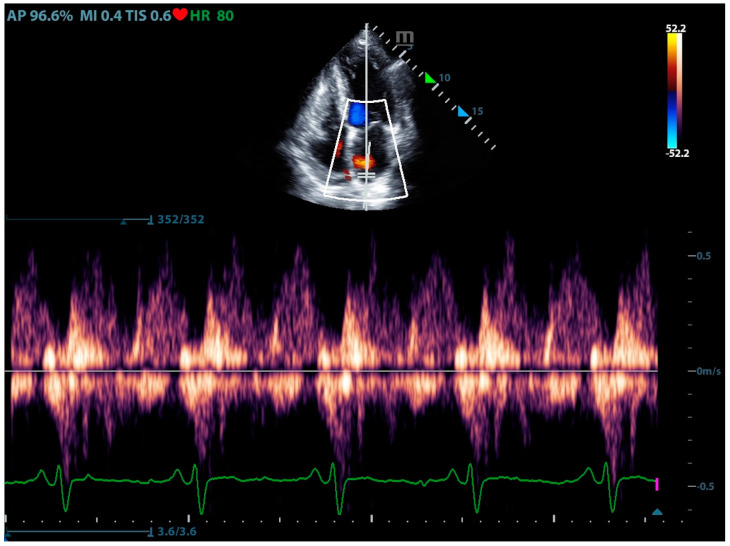
The ultrasound sign of pulmonary venous excess waveform.

The pulmonary veins are the terminus of pulmonary circulation, and blood from the pulmonary veins ultimately drains into the left atrium. Therefore, the core of pulmonary vein assessment lies in the assessment of left atrial pressure (LAP). The waveform of the pulmonary venous can reflect LAP indirectly. The typical pulmonary venous waveform comprises a systolic antegrade S wave peak (S wave), an early diastolic antegrade peak (D wave), and a late diastolic retrograde peak (Ar wave) (Figure 8). The S wave is primarily influenced by variations in left atrial pressure as well as left atrial contraction and relaxation. Meanwhile, the D wave predominantly reflects left ventricular compliance. Normally, the S wave is larger than the D wave. However, as the filling pressure of the left heart increases and the compliance of the left atrium and left ventricle decreases, the S/D ratio becomes less than 1, and a retrograde S wave may even manifest. The Ar wave is a negative wave generated when a portion of blood enters the pulmonary vein from the atrium during atrial contraction. It is impacted by left atrial contraction and the filling pressure of the left atrium and left ventricle. The velocity and duration of the Ar wave increase with rising left heart filling pressure [30,31].

The left atrial pressure (LAP) is intricately linked to the diastolic function of the left ventricle. In cases where the left ventricle develops II-III grade diastolic dysfunction, an elevation in left atrial pressure ensues. Diastolic dysfunction of the left ventricle is prevalent among critically ill patients and often precedes systolic dysfunction. With advancing age, individuals commonly experience a decline in diastolic function, initially characterized by a reduction in relaxation function. Virtually all cardiac diseases can induce some degree of diastolic dysfunction, and ultrasound detection of diastolic dysfunction typically reflects an advanced stage. Echocardiography enables rapid identification of diastolic dysfunction and provides a preliminary assessment through qualitative methods. Notably, patients with left ventricular hypertrophy frequently exhibit diastolic dysfunction, and those with atrial fibrillation are also prone to this condition due to irregular atrial contraction. Furthermore, when systolic dysfunction affects the left ventricle, its diastolic function is often concomitantly compromised. Enlargement of the left atrium is a prominent feature in these scenarios.

Left atrial pressure (LAP) is closely related to the diastolic function of the left ventricle. When the left ventricular develops II–III grade diastolic dysfunction, it can cause an increase in the left atrial pressure. Left ventricular diastolic dysfunction is very common in critically ill patients and often precedes systolic dysfunction. In the process of aging, humans often begin to experience a decline in diastolic function, which is initially manifested as a decrease in relaxation function. Nearly all cardiac diseases can cause some degree of diastolic dysfunction, and the diastolic dysfunction detected by ultrasound is already in a relatively late stage. Echocardiography can quickly identify whether a patient has diastolic dysfunction and allow a preliminary evaluation through qualitative methods. First, patients with left ventricular hypertrophy often have diastolic dysfunction. Second, patients with atrial fibrillation often also have diastolic dysfunction due to the lack of regular atrial contraction. In addition, when systolic dysfunction occurs in the left ventricle, its diastolic function is usually also affected. Enlargement of the left atrium is also a prominent feature in the above situations.

A variety of ultrasound indicators can be used to quantitatively evaluate left ventricular diastolic function at the bedside and help discover and evaluate the degree of diastolic dysfunction through pulse Doppler, tissue Doppler, color Doppler, and other modes. Mitral blood flow velocity is still the most used method to assess the diastolic function of the left ventricle. Using tissue Doppler to measure the early diastolic myocardial motion velocity e’ of the interventricular septum and the lateral wall can reflect the left ventricle relaxation function. In addition, the size of the left atrial and the regurgitation velocity of the tricuspid valve are also commonly used to assess left ventricular diastolic function. In 2016, the American Society of Echocardiography and the European Association of Cardiovascular Imaging proposed an assessment process for left ventricular diastolic function, which evaluated patients with normal systolic function and those with decreased systolic function or structural heart disease (Table 2) [13,14].

### 3.2. Ultrasound Assessment of the Lungs and Pulmonary Microcirculation Injuries

The systemic inflammation response of critical illness may lead to injuries of capillary endothelial cells and an increase in the permeability of capillaries, which may further cause fluid leakage of microcirculation and formation of extravascular edema [32]. There are a large number of capillary networks in the lungs. When pulmonary microcirculation injuries occur, the fluid of pulmonary microcirculation will leak into the pulmonary mesenchyme through the endothelial cells and aggregate into pulmonary edema. Fluid overload may cause an increase in the output of the right ventricle and increase in left atrial pressure, which exacerbate the injuries of pulmonary microvascular. As the disease progresses, the increase in extravascular pulmonary water may distribute to the gravity-dependent areas and form lung consolidation and atelectasis. 

The signs of lung ultrasound can help evaluate the degree of injury of the lungs and pulmonary microcirculation. Normal lung tissue may present as echogenic, gradually fading horizontal lines arranged at equal intervals below the pleural line (A-lines) at ultrasound scanning. When the air-water ratio is abnormal, vertical, laser-like echogenic lines extending from the pleural lines (B-lines) will appear. During the increase in the pulmonary interstitial edema and the decrease of air in the alveolus, the B-lines are increasing and becoming merged. When the lung tissue contains little or no air in the situation of consolidation or atelectasis, the ultrasound shows the echo density of the lung tissue similar to that of the liver or the spleen [33]. 

There are several semi-quantitative methods to assess the severity of lung and pulmonary microcirculation injury by lung ultrasound. The lung ultrasound score (LUS) is a scoring system based on the 12-region lung ultrasound scan method with a total score ranging from 0 to 36. The LUS system scores the severity of the lung injury based on the number of B-lines [34,35]. A modified LUS score was established that assessed the lung injury based on the percentage of involved pleural, which has better measurement performance. During the COVID-19 pandemic, four items including pleural effusion, pericardial effusion, inferior vena cava, and diaphragm displacement were added to establish the iLUS score based on the LUS score. The iLUS score is used to guide intensivists to evaluate the severity of COVID-19 pneumonia and guide further treatment [36]. The typical ultrasound signs and LUS scores are summarized in Table 3. 

### 3.3. Diaphragm Ultrasound and Respiratory Drive

The diaphragm is the main respiratory muscle. Ultrasound evaluation of diaphragm function can help explain the level of respiratory drive in critically ill patients and avoid spontaneous lung injury caused by inappropriately high respiratory drive [37]. Ultrasound evaluation of the diaphragm includes diaphragm excursion and diaphragm thickening fraction (TFdi).

Diaphragm excursion can be measured at the midclavicular line under the costal arch with the liver as the acoustic window. During spontaneous breathing, diaphragm excursion can reflect the patient’s respiratory drive and diaphragm function. However, diaphragm excursion is affected by positive pressure ventilation and cannot reflect the respiratory drive of patients with mechanical ventilation [38,39].

Diaphragm thickness can be measured at the midaxillary intercostal. Based on the end-inspiratory and end-expiratory diaphragm thickness, diaphragm thickness fraction (TFdi) can be calculated [40,41]. TFdi = (end-inspiratory diaphragm thickness-end-expiratory thickness)/end-expiratory thickness. Studies have shown that TFdi is consistent with the patient’s respiratory effort and is considered to be one of the indicators for quantitative evaluation of respiratory drive in mechanical ventilation patients [42,43]. Goligher et al. found that TFdi levels of 15–30% were associated with the least total mechanical ventilation duration in patients [44]. Therefore, appropriately preserving the spontaneous breathing of the diaphragm while avoiding high respiratory drive is conducive to promoting the weaning and rehabilitation of critically ill patients.

Diaphragm velocity can be measured using tissue Doppler technology. Diaphragmatic peak systolic velocity (PCV) is correlated with the peak trans-diaphragmatic pressure (peakPdi) and trans-diaphragmatic pressure-time integral (PTPdi) obtained by esophageal manometry and is a reliable indicator of respiratory drive in critically ill patients [45]. The application of ultrasound tissue Doppler measurement of the diaphragm to guide the setting of mechanical ventilation in critically ill patients is one of the hot spots of further research.

#### The Protocol of Critical Point-of-Care Ultrasound Pulmonary Circulation Assessment

**Figure 9:**
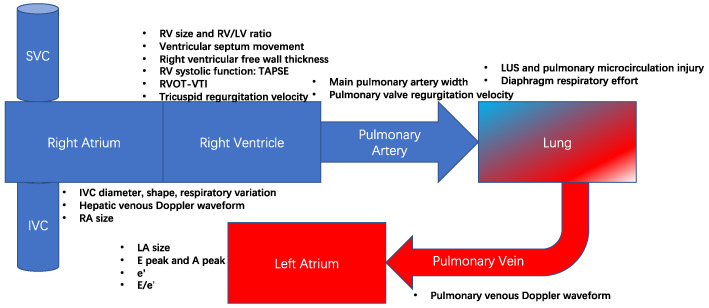
Summary of point-of-care ultrasound evaluation protocol of pulmonary circulation. Abbreviations: SVC, superior vena cava; IVC, inferior vena cava; RV, right ventricle; LV, left ventricle; TAPSE, tricuspid annular plane systolic excursion; RVOT, right ventricular outflow tract; VTI, velocity-time integral; LUS, lung ultrasound; LA, left atrium.

Bedside critical care ultrasound not only assesses the pressure and flow of pulmonary circulation but also provides information related to pulmonary circulation such as the severity of lung injury and respiratory drive. More importantly, lung ultrasound can also provide evidence of pulmonary microcirculation damage, making the assessment of pulmonary circulation more comprehensive. We developed a point-of -care ultrasound protocol for a comprehensive assessment of pulmonary circulation in the order of the direction of blood flow, including IVC, right ventricle, right atrium, pulmonary artery, lung, pulmonary vein, and left atrium, which is shown Figure 9.

## 4. The Role of Critical Care Ultrasound in Critical Illnesses Involving Pulmonary Circulation

### 4.1. Acute Respiratory Distress Syndrome

Acute respiratory distress syndrome (ARDS) is a common critical illness with high morbidity and mortality. During the development of various diseases such as sepsis, shock, trauma, and burns, the damage of pulmonary capillary endothelial cells and alveolar epithelial cells causes diffuse pulmonary interstitial and alveolar edema, leading to acute hypoxic respiratory insufficiency or respiratory failure [9,46]. ARDS not only causes lung injury but also damages pulmonary circulation. Bedside lung ultrasound can assess the lesions in different regions of ARDS patients and quantify the severity of injury based on the LUS score [20,34,35]. Lung ultrasound can also guide the selection of lung recruitment and positional therapy based on the distribution and severity of lung injury, evaluate the effect of treatment, and detect complications such as pneumothorax during treatment [47].

In patients with ARDS, restrictive fluid management strategies can help improve oxygenation and reduce pulmonary circulation injury [46,48,49,50]. ARDS patients are prone to acute cor pulmonale (ACP) when the plateau pressure and driving pressure are too high even with a lung protective ventilation strategy. Studies have shown that the incidence of ACP in ARDS patients is as high as 25%, while the mortality rate of ARDS combined with ACP is as high as 60% [51,52]. The risk factors of ACP in patients with ARDS include inflammatory lung injury, driving pressure > 18 cm H_2_O, PaO_2_/FiO_2_ < 150 mmHg, and PaCO_2_ > 48 mmHg [3]. It is necessary to implement a pulmonary circulation protection strategy based on right heart protection, which includes controlling plateau pressure, reducing driving pressure, and avoiding hypercapnia, to reduce pulmonary circulation resistance and reduce right heart afterload. During this process, hemodynamic assessment using critical care ultrasound is an essential part of pulmonary circulation management in ARDS patients [53,54].

### 4.2. Sepsis

Sepsis is defined as life-threatening organ dysfunction caused by a dysregulated host response to infection [55]. Although the initial conditions of and underlying causes in patients vary, all patients are in a high metabolic state due to the activation of the immune system and the release of inflammatory factors [56]. Reduced peripheral vascular tone and excessive release of catecholamine in sepsis patients lead to a hyperdynamic state of the heart [57]. Studies have shown that a prolonged cardiac hyperdynamic state can lead to septic cardiomyopathy, leading to a poor overall prognosis for patients [58,59,60]. Critical care ultrasound can rapidly assess the systolic function of the heart by eyeballing, which has a high correlation with indicators of left ventricular systolic function such as LVEF and MAPSE [61]. Critical care cardiac ultrasound can be used to rapidly assess the state of the heart, evaluate the response to anti-stress treatment in a timely manner, and protect cardiac function.

The systemic inflammatory response and excessive release of catecholamines caused by sepsis can lead to excessive degradation of the vascular endothelial glycocalyx, causing damage to vascular endothelial cells [62,63]. Destruction of pulmonary capillary endothelial cells leads to increased pulmonary capillary permeability, which in turn leads to pulmonary interstitial edema and gravity-dependent consolidation [46]. The signs of lung ultrasound can quickly assess the extent and degree of pulmonary microcirculatory damage [47].

Sepsis can cause damage to the heart muscle, resulting in septic cardiomyopathy [64]. Septic cardiomyopathy can be divided into five subtypes based on echocardiographic findings: LV systolic dysfunction, LV diastolic dysfunction, RV dysfunction, diffuse cardiac dysfunction, and mixed [65]. Right ventricular failure tends to occur in patients with sepsis who are under mechanical ventilation, combined with ARDS and other factors that cause increased right ventricular afterload [66]. Critical care ultrasound can rapidly assess RV preload, afterload, and systolic function of the right ventricle and provide early detection of right ventricular dysfunction. Reducing RV preload and afterload is beneficial for preserving RV function in patients with sepsis to ensure the perfusion of organs and tissues throughout the body. Critical care ultrasound is useful to guide the titration of right ventricular preload and afterload and to optimize right heart management in patients with sepsis [67].

### 4.3. Pulmonary Embolism (PE) and Pulmonary Hypertension

Pulmonary embolism (PE) is a condition in which an embolus from the venous system or the right heart blocks the pulmonary artery or its branches. Depending on the extent of the PE and the underlying cardiorespiratory status, the right heart is affected to varying degrees. Excessive right heart pressure causes a decrease in left ventricular filling, resulting in a decrease in left ventricular cardiac output and systemic ischemia. Severe cases may result in refractory hypotension and cardiac arrest [68]. Echocardiography evaluates right heart function and pulmonary circulation, which can help guide the diagnosis and treatment of pulmonary embolism.

The immediate sign of pulmonary embolism is an embolus in the pulmonary artery or right heart system. Compared with transthoracic echocardiography (TTE), transesophageal echocardiography (TEE) can observe the right heart and pulmonary artery structure, and it is easier to find the right heart and pulmonary artery emboli [69]. TTE can reflect the increase in right heart pressure due to right ventricular dilatation, the contradictory motion of the interventricular septum, and dilatation and fixation of the inferior vena cava. TAPSE reflects the long-axis systolic function of the right heart. TAPSE > 15 mm indicates normal right heart contraction and <8 mm indicates right heart systolic dysfunction. In the early stages of pulmonary embolism, right heart systolic function may be temporarily increased to compensate for increased right heart pressure. As the disease progresses to an advanced stage, right ventricular function decompensates and right ventricular systolic function may decline [70]. Ultrasound estimation of pulmonary artery pressure from tricuspid regurgitation velocity is also used to guide the diagnosis and treatment of pulmonary embolism.

### 4.4. Pulmonary Congestion and Edema

Acute pulmonary congestion and edema, characterized by the exudation of fluid from pulmonary capillaries into the pulmonary parenchyma and alveoli, leads to impaired gas exchange. There are two categories of clinical pulmonary congestion and edema. The first category involves acute decompensated heart disease resulting in increased pulmonary capillary hydrostatic pressure, leading to the over-production of extravascular pulmonary water, ultimately causing pulmonary edema. The second category encompasses pulmonary fluid leakage caused by increased permeability of pulmonary capillaries due to other diseases such as acute lung injury, sepsis, and allergy. Pulmonary circulation congestion and pulmonary edema result from a mismatch between left and right ventricular stroke volume, where increased hydrostatic pressure in the pulmonary capillaries occurs when left ventricular ejection is decreased or right ventricular ejection is increased. Therefore, an ultrasound evaluation of both the left and right heart is essential to guide the treatment of cardiogenic pulmonary edema.

The formation of left-sided pulmonary edema is mainly due to the increase in left atrial pressure. The peak flow velocity (E peak, A peak) in the early and late phases of mitral diastole, mitral valve e’, and pulmonary venous blood flow spectrum can be used to estimate the left atrial pressure level and dynamically guide dehydration treatment [11,14]. Right heart involvement is more common in critically ill patients. When right heart preload and afterload increase for various reasons, right heart output increases. When the cardiac output of the left heart does not match that of the right heart, pulmonary edema increases [71]. Assessment of the IVC, right ventricular size and motion, and RVOT-VTI are all useful in the management of pulmonary edema.

## 5. Conclusions and Perspective

Pulmonary circulation is an important part of the circulation system. It plays a vital role in the process of the transportation of blood flow and oxygen flow. The unique physiological characteristics of pulmonary circulation make each component of it vulnerable to involvement during the progression of critical illnesses. This susceptibility is due to the direct impact of the primary disease and the secondary systemic inflammatory response. In ICU, some inappropriate interventions like mechanical ventilation and fluid overload may increase the preload and afterload of the right heart and exacerbate the involvement of pulmonary circulation. Point-of-care ultrasound enables visual evaluation of each component of pulmonary circulation in the blood flow direction. Qualitative and quantitative ultrasound assessment helps rapidly identify pulmonary circulation involvement in critically ill patients, determine the causes of pulmonary circulation involvement, and guide adjustments to ICU treatment.

However, there are limitations in the assessment of pulmonary circulation using point-of-care ultrasound. Firstly, mastering ultrasound technology requires periodic training for ICU physicians. Additionally, factors such as positioning, surgical procedures, and catheters make it difficult to obtain standard views and angles for measuring ultrasound parameters, especially for beginners. In addition, point-of-care ultrasound cannot replace other monitoring methods. For example, central venous pressure measurement via internal jugular vein catheterization still plays an important role as a simple and highly repeatable method in the ICU. Furthermore, pulse contour cardiac output (PiCCO) and pulmonary artery catheter (PAC) can provide more precise hemodynamic information. Combining qualitative and quantitative ultrasound measurements with these hemodynamic parameters can more accurately explain the pathophysiological changes in pulmonary circulation of critically ill patients and guide subsequent treatment.

The integration of artificial intelligence (AI) technology in medical practice has become increasingly prevalent. In the context of pulmonary circulation ultrasound assessment, AI technology is anticipated to significantly influence image quality control, parameter interpretation, and educational practices. Moreover, the utilization of wearable ultrasound devices for continuous, real-time, and dynamic measurements is expected to substantially augment the volume of data within AI training sets, consequently enhancing AI performance. These developments are poised to emerge as pivotal areas of focus in future research endeavors.

## Figures and Tables

**Table 1:** Ultrasound appearance of the inferior vena cava predicts right atrial pressure for spontaneous breathing patients.

IVC Diameter	IVC Collapse Rate	RAP/CVP
>21 mm	<50%	15 mmHg
<21 mm	>50%	3 mmHg
Between the two above		8 mmHg

Abbreviations: IVC, inferior vena cava; RAP, right atrial pressure; CVP, central venous pressure.

**Table 2:** Criteria of left ventricular diastolic dysfunction.

LVEF	Criteria
Normal LVEF	At least 3 of the following criteria are met: ① e’ velocity (e’ < 7 cm/s on the septal annulus; e’ < 10 cm/s on the lateral annulus) ② E/e’ > 14 (lateral annulus E/e’ > 13, septal annulus E/e’ > 15) ③ Left atrial volume index > 34 mL/m^2^ ④ Tricuspid regurgitation peak velocity > 2.8 m/s
Reduced LVEF	① E/A ≥ 2 ② 0.8 < E/A < 2, peak E velocity > 50 cm/s and 2 of the following criteria are met: Mean E/e’ > 14; Left atrial volume index > 34 mL/m^2^ Tricuspid regurgitation velocity > 2.8 m/s

Abbreviation: LVEF, left ventricular ejection fraction.

**Table 3:** Lung ultrasound signs and pulmonary microcirculation injuries.

Ultrasound Sign	Pathological Change	LUS Score	Image
A-lines	Normal lung tissue	0	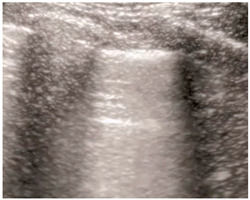
B-lines involved <50% of the pleural	Early stage of pulmonary endothelial injury	1	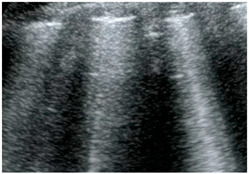
B-lines involved >50% of the pleural	Pulmonary interstitial edema, endothelial and epithelial injury	2	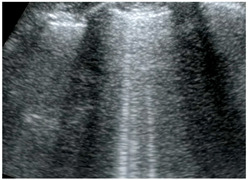
Consolidation	Water consolidation in gravity-dependent areas	3	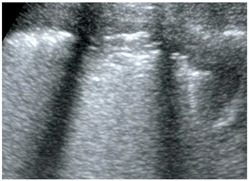

Abbreviation: LUS, lung ultrasound.

## Data Availability

No new data were created.
